# Knowledge of the health risks of smoking and impact of cigarette warning labels among tobacco users in six European countries: Findings from the EUREST-PLUS ITC Europe Surveys

**DOI:** 10.18332/tid/99542

**Published:** 2019-02-13

**Authors:** Antigona C. Trofor, Sophia Papadakis, Lucia M. Lotrean, Cornel Radu-Loghin, Marius Eremia, Florin Mihaltan, Pete Driezen, Christina N. Kyriakos, Ute Mons, Tibor Demjén, Sarah O. Nogueira, Esteve Fernández, Yannis Tountas, Krzysztof Przewoźniak, Ann McNeill, Geoffrey T. Fong, Constantine I. Vardavas, on behalf of the EUREST-PLUS consortium

**Affiliations:** 1 University of Medicine and Pharmacy, ‘Grigore T. Popa’ Iasi, Iasi, Romania; 2 Aer Pur România, București, România; 3 University of Crete (UoC), Heraklion, Greece; 4 Division of Prevention and Rehabilitation, University of Ottawa Heart Institute, Ottawa, Canada; 5 Iuliu Hatieganu University of Medicine and Pharmacy, Cluj-Napoca, Romania; 6 European Network for Smoking and Tobacco Prevention (ENSP), Brussels, Belgium; 7 University of Medicine and Pharmacy ‘Carol Davilla’, Bucharest, Romania; 8 Department of Psychology, University of Waterloo (UW), Waterloo, Canada; 9 Cancer Prevention Unit and WHO Collaborating Centre for Tobacco Control, German Cancer Research Center (DKFZ), Heidelberg, Germany; 10 Smoking or Health Hungarian Foundation (SHHF), Budapest, Hungary; 11 Tobacco Control Unit, Catalan Institute of Oncology (ICO), Barcelona, Spain; 12 Cancer Control and Prevention Group, Bellvitge Biomedical Research Institute, Barcelona, Spain; 13 National and Kapodistrian University of Athens (UoA), Athens, Greece; 14 Health Promotion Foundation (HPF), Warsaw, Poland; 15 Maria Skłodowska-Curie Institute-Oncology Center (MSCI), Warsaw, Poland; 16 National Addiction Centre, Addictions Department, Institute of Psychiatry, Psychology & Neuroscience, King’s College London (KCL), London, United Kingdom; 17 UK Centre for Tobacco and Alcohol Studies, London, United Kingdom; 18 School of Public Health and Health Systems, University of Waterloo (UW), Waterloo, Canada; 19 Ontario Institute for Cancer Research, Toronto, Canada

**Keywords:** tobacco use knowledge, health effects, health warnings, Europe, ITC

## Abstract

**INTRODUCTION:**

The aim of this study was to examine knowledge of health effects of smoking and the impact of cigarette package warnings among tobacco users from six European Union (EU) Member States (MS) immediately prior to the introduction of the EU Tobacco Products Directive (TPD) in 2016 and to explore the interrelationship between these two factors.

**METHODS:**

Cross-sectional data were collected via face-to-face interviews with adult smokers (n=6011) from six EU MS (Germany, Greece, Hungary, Poland, Romania, Spain) between June–September 2016. Sociodemographic variables and knowledge of health risks of smoking (KHR) were assessed. Warning salience, thoughts of harm, thoughts of quitting and foregoing of cigarettes as a result of health warnings were assessed. The Label Impact Index (LII) was used as a composite measure of warning effects. Linear and logistic regression analyses were used to examine sociodemographic predictors of KHR and LII and the inter-relationship between knowledge and LII scores.

**RESULTS:**

The KHR index was highest in Romania and Greece and lowest in Hungary and Germany. While the majority of smokers knew that smoking increases the risk for heart diseases, lung and throat cancer, there was lower awareness that tobacco use caused mouth cancer, pulmonary diseases, stroke, and there were very low levels of knowledge that it was also associated with impotence and blindness, in all six countries. Knowledge regarding the health risks of passive smoking was moderate in most countries. The LII was highest in Romania and Poland, followed by Spain and Greece, and lowest in Germany and Hungary. In almost all countries, there was a positive association between LII scores and higher KHR scores after controlling for sociodemographic variables. Several sociodemographic factors were associated with KHR and LII, with differences in these associations documented across countries.

**CONCLUSIONS:**

These data provide evidence to support the need for stronger educational efforts and policies that can enhance the effectiveness of health warnings in communicating health risks and promoting quit attempts. Data will serve as a baseline for examining the impact of the TPD.

## INTRODUCTION

The World Health Organization Framework Convention on Tobacco Control (WHO FCTC), an unprecedented international treaty aimed at strengthening tobacco control globally, underlines the importance of education, communication, training, and public awareness on the health risks of smoking^1^. A key aspect to educating the public on the deleterious effects of tobacco is the adoption of health warnings on cigarette packages^1,2^. Article 11 of the WHO FCTC addresses packaging and labeling of tobacco products by calling for ‘each unit packet and package of tobacco products, and any outside packaging and labeling of such products, to carry health warnings describing the harmful effects of tobacco use, and include other appropriate messages’^1^. With the exception of television advertisements, health warnings on cigarette packages are among the most effective sources of information regarding the health effects of smoking, with most smokers reporting becoming more aware about the risks of smoking through warnings on cigarette packages compared to other sources of information^2^. Evidence shows that combined written and graphic health messages are more effective than text-only warnings^2-4^. Pictorial warnings have been shown to increase levels of knowledge regarding the health effects of smoking, as well as increase motivation for smoking cessation behavior among smokers^2-9^.

The updated European Union (EU) Tobacco Products Directives (TPD), which took effect in May 2016, is a major effort to propel and standardize the ratification of the WHO FCTC among EU Member States (MS), and has reinforced the importance of warning labels as an important tobacco control strategy since they are compelling communication strategy, combining high exposure, high reach, and very low cost^10-12^. In consideration of the strong evidence that health warnings increase awareness of health risks related to tobacco consumption, increase quit attempts and decrease smoking uptake and the realization of the disparities that exist among the EU MS in the implementation of the FCTC, the EU TPD has set forth rules governing the parameters of health warning labels that are scientifically supported to maximize impact. The TPD effectively requires combined text and pictorial health warnings that cover 65% of the front and back of tobacco product packaging^10-12^.

In many EU countries, full implementation of the 2016 TPD occurred in late fall of 2017. Prior to the TPD coming into effect, EU MS varied in the extent to which they had implemented text and pictorial warnings (Table 1). Moreover, the messages transmitted through health warnings varied across countries (Table 2).

**Table 1:** Description of tobacco product warning labels in countries sampled in 2016^1^

*Country*	*Text warnings only*	*Medium (<50% of the front and back of package) pictorial warnings*	*Large (≥50% of the front and back of package) pictorial warnings*
Germany	X		Introduced May 2016^2^
Greece	X		
Hungary		X	Introduced August 2016^3^
Poland	X		Introduced May 2016^2^
Romania		X	
Spain			X^4^

1 Information refers to the data collection period (June–September 2016).

2 Introduced May 2016, but slow market penetration due to stockpiling.

3 Introduced on small quantity of packs in market.

4 Even though the TPD was not transposed in Spain at the time of the fieldwork, several manufacturers already sold packages according to the TPD requirements.

**Table 2:** Content of health warnings in 2016 by country and Tobaccco Product Directive (TPD)^1^

*Health issue^2^*	*Germany*	*Greece*	*Spain*	*Romania*	*Hungary*	*Poland*	*TPD^3,4^*
**Active smoking**							
Heart disease	Yes	Yes	Yes	Yes	Yes	Yes	Yes
Impotence	Yes	Yes	Yes	Yes	Yes	Yes	Yes
Lung cancer	Yes	Yes	Yes	Yes	Yes	Yes	Yes
Blindness	No	No	No	No	No	No	Yes
Mouth cancer	No	No	No	No	No	No	Yes
Throat cancer	No	No	Implied^5^	Implied^5^	Implied^5^	No	Yes
Stroke	Yes	Yes	Yes	Yes	Yes	Yes	Yes
Emphysema	Implied^6^	Implied^7^	Implied^7^	Implied^7^	Implied^7^	Implied^6^	Implied^8^
Bronchitis	Implied^6^	Implied^7^	Implied^7^	Implied^7^	Implied^7^	Implied^6^	Implied^8^
**Passive smoking**							
Lung cancer	No	Implied^9^	No	No	No	No	Implied^10^
Heart attacks	No	Implied^9^	No	No	No	No	Implied^10^
Asthma in children	Implied^11^	Implied^11^	Implied^12^	Implied^12^	Implied^12^	Implied^12^	Implied^10^

1 Information is provided for the content of the health warnings in June-September 2016 during the period of survey.

2 Information is provided for the content of the health warnings with regard to the issues included in the questions regarding the knowledge regarding health risks of smoking investigate by the survey.

3 TPD was in the process of introduction during the survey period in Germany, Hungary and Poland, but only of small quantities of packages were on the market.

4 In Spain, even though the TPD was not oficially in place, the TPD health warnings were already found on the packages of several brands on the market.

5 Smoking can cause a painful slow death, with a pictogram showing a tumour at the level of the throat.

6 Stopping smoking reduces the risk of serious heart and lung disease.

7 Quiting smoking decreases your risk for cardiovascular and pulmonary diseases.

8 Smoking damages your lungs.

9 Smoking seriously harms you and the ones around you.

10 Smoking harms your children, family and friends.

11 Protect children, do not force them to breathe in your smoke.

12 Protect your child, do not let them breathe your smoke. Smoking during pregnancy is bad for your child.

In order to evaluate the impact of the TPD provisions on health warnings, it is necessary to establish a better understanding of the knowledge, perceptions, and behaviors related to heath warning labels among EU MS prior to the full implementation of such measures and establishment of a baseline for examining changes over time. This paper reports on knowledge of smoking health harms and the perceived impact of cigarette warning labels among smokers from six EU MS (Germany, Greece, Hungary, Poland, Romania, Spain) before the TPD exerted its full effects, and explores the interrelationship between these two factors as well sociodemographic variables.

## METHODS

### Study sample and methodology for data collection

The current study is part of a European Commission Horizon-2020 funded study entitled *European Regulatory Science on Tobacco: Policy implementation to reduce lung diseases* (EUREST-PLUSHCO-06-2015). The EUREST-PLUS Project aims to monitor and evaluate the impact of the implementing Acts of the TPD within the context of the FCTC and its implementation within Europe^13^. Using the methodology of The International Tobacco Control Policy Evaluation (ITC) Project, a cross-sectional survey was conducted among six European countries (Wave 1 6E)^14^. A nationally representative sample of 6011 cigarette smokers of age 18 years or older was collected from Germany (n=1003), Greece (n=1000), Hungary (n=1000), Poland (n=1006), Romania (n=1001), and Spain (n=1001). Ethics approval for data collection was provided for each national cohort by the corresponding local ethics committees and informed consent was received from all participants.

Data collection occurred between June 2016 and September 2016. According to Nomenclature of Territorial Units for Statistics (NUTS), the geographic strata were NUTS2 regions (NUTS1 in Germany) crossed with degree of urbanization (urban, semi-urban, rural). Approximately 100 area clusters were sampled in each country, with the aim of obtaining 10 adult smokers per cluster. Clusters were allocated to strata proportionally to the population size for adults 18 years and older. Within each cluster, household addresses were sampled using a random walk design. One randomly selected male smoker and one randomly selected female smoker were chosen for interview from a sampled household where possible. Screening of households continued until the required number of smokers from the cluster had been interviewed. All interviews were conducted face-to-face by interviewers using tablets. Further details on the methodology of the 6E Survey can be found elsewhere^13^.

During the data collection period (June–Sept. 2016), Greece had text warnings on tobacco packages, while Germany and Poland had just moved from text warnings to large pictorial warnings covering ≥50% of the front and back of the package in May 2016. Romania, Hungary and Spain had medium-size pictorial warning (<50% of the front and back of package) which had been in place since 2008 (Romania) or 2011 (Hungary and Spain); in Hungary since 20 August 2016, there may have been tobacco products with large pictorial warnings, but compared to all, probably only to a negligible extent (Table 1).

### Measures

#### Knowledge of health risks

In order to assess knowledge of the health risks of smoking, respondents were presented with nine diseases and conditions (heart disease, impotence, lung cancer, blindness, mouth cancer, throat cancer, stroke, emphysema, bronchitis) and were asked to state whether they believed the condition is caused by smoking. Respondents were also asked to state if they believed secondhand smoke caused lung cancer in non-smokers, heart attacks in non-smokers, and asthma in children. Knowledge of health risks was coded as 0=‘not caused by smoking/don’t know’ and 1=‘caused by smoking’. An index regarding knowledge of the health risks of smoking (KHR) was calculated by summing the standardized scores (z-scores calculated across all countries) of each participant’s responses to the questions pertaining to health risks of smoking.

#### Warning label impact

Warning label effects were measured using six indicators as in previous ITC surveys^7,8,15-16^. Each indicator was analyzed as a dichotomous measure of frequency. Warning label *salience* was assessed using two questions: 1) NOTICING: ‘in the last month, how often, if at all, have you noticed the warning labels on cigarette packages (1=very often or often; 0=sometimes, rarely, or never)?’, and 2) READING: ‘In the last month, how often, if at all, have you read or looked closely at the warning labels on cigarette packages (1=often; 0=never or once in a while)?’. THOUGHTS of harm were assessed with the following question: ‘to what extent, if at all, do the warning labels make you think about the health risks of smoking (1=a lot; 0=somewhat, a little, or not at all)?’. Thoughts of QUITTING were assessed with the question: ‘to what extent, if at all, do the warning labels on cigarette packs make you more likely to quit smoking (1=a lot; 0=somewhat, a little, or not at all)?’. FORGOING of cigarettes was assessed by asking: ‘in the last month, have the warning labels stopped you from having a cigarette when you were about to smoke one (1=many times, a few times, or once; 0=never)?’. AVOIDING was assessed: ‘in the last month, have you made any effort to avoid looking or thinking about the warning labels, such as covering them up, keeping them out of sight, using a cigarette case, avoiding certain warnings, or any other means (1=yes, 0=no)?’.

The Labels Impact Index (LII) was calculated using the methodology employed in several previous ITC reports^7,8,15-17^. The LII is a composite measure that combines four of the six warning label effectiveness indicators and then weighting and summing the standardized scores (z scores) using the following calculation: LII = (NOTICING × 1) + (THOUGHTS × 2) + (QUITTING × 2) + (FORGOING × 3). The LII was calculated using the original four/five-point scales of the individual measures of health warning effectiveness, not dichotomized. Higher LII scores indicate greater impact^16^.

Respondents were also asked if they would like to have more, less or the same amount of information contained in the health warnings.

#### Demographics and smoking behavior

Sociodemographic characteristics including country, age, gender, degree of urbanization (urban, semi-urban, rural), level of education (low, medium, high) and income level (low, medium, high) were assessed. Highest level of formal education completed categorized as: low (primary, lower pre-vocational secondary, middle pre-vocational secondary), moderate (secondary vocational, senior general secondary and pre-university) and high (higher professional and university Bachelor, university Masters). Due to the different income cut-off points across countries, income was categorized as low, medium, and high using standardized country-level cut-offs established by the ITC Project^18^ as follows: low income level (<€1750 for Germany, Greece and Spain; ≤150000 Ft for Hungary; ≤2000 zł for Poland; ≤1000 lei for Romania), moderate income (€1750 to €3000; 150001 Ft to 250000 Ft; 2001 zł to 4000 zł; 1001 lei to 2500 lei), and high income (>€3000, >250000 Ft, >4000 zł, >2500 lei). Smoking behavior included the assessment of number of cigarettes smoked per day (<10, 11–20, 21–30, >30).

### Statistical analyses

Data were analyzed with SPSS 20.0 using the Complex Samples package to account for the complex sampling design. We analyzed data for each country separately and calculated percentages and standard errors. All statistical estimates presented are weighted for sex, age and degree of urbanization to make the data more representative of the population of smokers in each country. Participants who did not offer information regarding sociodemographic characteristics or the variables assessing the knowledge about health risks of smoking or the warning label impact were excluded from the analyses. Linear regression analyses were conducted to examine predictors of KHR and LII, the independent variables being age, gender, degree of urbanization, income, and education level, as well as the number of cigarettes smoked per day; these approaches were used based on previous relationships tested in other ITC studies^4,8,15^.

In order to assess if the label impact index influences knowledge regarding the health risks of smoking, two approaches were used. First, linear regressions were performed with the KHR index as the dependent variable and the LII index as the independent variable, controlling for sociodemographic variables, as well as the number of cigarettes smoked per day. Second, logistic regression was performed using as dependent variables the measures that assess the knowledge of the risk of smoking for several diseases, and using the LII index as the independent variable, controlling for age, gender, degree of urbanization, income, education level (sociodemographic variables) as well as the number of cigarettes smoked per day.

## RESULTS

### Characteristics of the study sample

The study sample consisted of 6011 persons (47.2% women and 52.8% men; 35.4% were from urban areas, 37.5% from semi-urban and 27.1% from rural areas). The mean age was 45.1 years (8.4%, 18–24 years; 29.5%, 25–39; 33.3%, 40–54; 28.7% older than 54 years). The education level was: 36.8% low, 51.6% medium, 11% high, 0.6% did not declare. With regard to the income, 22.2% had low income, 37.4% medium, 17.4% high income, while 23% did not declare.

### Knowledge of health risks (KHR) of smoking

With the exception of Hungary, more than 80% of the smokers in each country knew that active smoking causes heart diseases, lung cancer, and throat cancer (Table 3). More than two-thirds of smokers from all countries were aware of the risk of active smoking for pulmonary diseases (emphysema, bronchitis) and mouth cancer. In Spain and Hungary less than 60% of the smokers were aware of the risk of active smoking for stroke. The effect of smoking on impotence was recognized by approximately half of smokers and on blindness by one-third or less, the only exception being Romania where almost two-thirds knew the risk of smoking for impotence and more than half knew that smoking can cause blindness. Knowledge regarding the health risks of passive smoking was moderate in most countries, with lower levels of knowledge documented in Germany and Hungary. The risks of passive smoking were best known by smokers in Romania (Table 3). Smokers from Hungary and Germany also reported the lowest KHR index score, while the highest scores were documented in Greece and Romania.

**Table 3:** Knowledge of the health risks (KHR) of smoking overall and by country (n=6011)

*Health Effect*	*Overall*	*Germany*	*Greece*	*Spain*	*Romania*	*Hungary*	*Poland*
	*Per cent who agreed (SE)*						
**Active smoking**							
Heart disease	82.7 (1.0)	81.9 (2.8)	94.9 (1.1)	81.9 (2.5)	87.7 (1.6)	67.0 (3.1)	82.7 (2.6)
Impotence	56.1 (1.3)	50.2 (3.5)	61.7 (3.0)	53.7 (2.9)	64.3 (2.6)	54.7 (3.3)	52.0 (3.3)
Lung cancer	90.0 (0.7)	89.8 (2.6)	96.1 (0.8)	93.1 (1.8)	90.2 (1.2)	83.4 (1.9)	87.4 (1.9)
Blindness	34.7 (1.2)	23.8 (2.6)	33.0 (3.1)	24.4 (3.2)	57.6 (2.8)	35.7 (3.3)	33.9 (2.8)
Mouth cancer	78.0 (0.9)	71.3 (3.0)	85.3 (1.6)	84.7 (2.0)	76.7 (2.0)	70.7 (2.6)	79.3 (2.4)
Throat cancer	83.1 (0.9)	82.3 (2.8)	88.0 (1.4)	78.5 (2.3)	88.6 (1.3)	76.7 (2.3)	84.8 (2.2)
Stroke	65.2 (1.1)	71.5 (3.2)	68.6 (2.6)	56.4 (2.9)	78.1 (1.8)	55.6 (3.0)	61.0 (2.9)
Emphysema	72.5 (1.1)	68.7 (2.9)	78.6 (2.1)	75.2 (2.5)	66.2 (3.1)	69.1 (2.8)	77.2 (2.8)
Bronchitis	79.5 (1.0)	77.4 (2.7)	88.4 (1.7)	92.2 (1.7)	80.8 (1.7)	68.2 (3.0)	69.7 (3.0)
**Secondhand smoke**							
Lung cancer	68.2 (1.2)	58.4 (3.4)	70.1 (3.1)	67.3 (3.6)	82.3 (1.7)	57.9 (3.2)	72.8 (2.7)
Heart attacks	56.3 (1.3)	41.1 (3.4)	63.2 (3.2)	51.3 (3.7)	73.4 (2.4)	43.5 (3.5)	65.1 (3.0)
Asthma in children	68.5 (1.2)	59.8 (3.6)	73.8 (2.8)	68.2 (2.7)	78.9 (2.0)	63.9 (3.2)	66.5 (2.8)
	**Mean (SE)**						
**KHR Index^a^**	-0.046 (0.220)	-1.154 (0.662)	1.614 (0.371)	-0.110 (0.514)	1.868 (0.435)	-2.029 (0.610)	0.002 (0.583)

SE: standard error.

a KHR Index, calculated using z scores

The results of the regression analysis examining predictors or KHR are reported in Table 4. KHR index scores were lower among men than women in Germany, Hungary and Poland. In Romania and Greece, significantly lower KHR index scores were documented for smokers of age 18–24 years, compared to older age groups. In Poland, smokers who smoke less cigarettes/day had a better score. No other significant associations were documented for income or education at the country-level.

**Table 4:** Predictors of knowledge of the health risks (KHR) of smoking index (results of linear regression analysis)

*Variable*	*Germany*	*Greece*	*Spain*	*Romania*	*Hungary*	*Poland*
	*B (95% CI)*					
**Age group**						
18–24	0.45	-2.72*	0.53	-3.29**	-2.63	0.07
	(-2.19, 3.08)	(-5.24, -0.20)	(-3.05, 4.12)	(-5.35, -1.22)	(-7.16, 1.90)	(-2.76, 2.90)
25–39	0.57	-0.01	-0.205	-2.02**	-1.08	-1.12
	(-1.60, 2.74)	(-1.03, 1.01)	(-2.61, 2.20)	(-3.45, -0.58)	(-3.72, 1.55)	(-3.24, 0.99)
40–54	1.37	0.06	0.88	0.59	-0.27	0.58
	(-0.68, 3.42)	(-1.15, 1.27)	(-0.39, 2.15)	(-0.68, 1.88)	(-2.46, 1.92)	(-1.11, 2.27)
> 54	Ref	Ref	Ref	Ref	Ref	Ref
**Gender**						
Male	-1.59***	-0.49	0.19	-0.06	-1.64*	-1.30*
	(-2.50, -0.68)	(-1.46, 0.48)	(-1.49, 1.90)	(-1.56, 1.45)	(-3.01, -0.28)	(-2.38, -0.22)
Female	Ref	Ref	Ref	Ref	Ref	Ref
**Urbanization degree**						
Urban	0.50	0.19	-0.23	0.63	1.60	0.47
	(-2.92, 3.93)	(-2.12, 2.51)	(-2.45, 2.40)	(-1.39, 2.66)	(-2.58, 5.79)	(-2.30, 3.24)
Semi-urban	1.23	1.28	0.40	1.30	2.66	-1.280
	(-1.30, 3.95)	(-0.63, 3.19)	(-2.13-2.93)	(-1.10, 3.70)	(-1.40, 6.73)	(-4.04, 1.48)
Rural	Ref	Ref	Ref	Ref	Ref	Ref
**Education**						
Low	-2.18	-0.96	-1.60	-0.65	1.09	-1.32
	(-5.1, 0.64)	(-2.52, 0.59)	(-3.70, 0.52)	(-3.56, 2.25)	(-3.43, 5.62)	(-4.21, 1.57)
Medium	-0.78	0.57	-1.38	-0.72	0.46	-0.258
	(-2.94, 1.38)	(-0.72, 1.86)	(-3.28, 0.52)	(-2.43, 0.98)	(-4.07, 4.99)	(-2.44, 1.93)
High	Ref	Ref	Ref	Ref	Ref	Ref
**Income**						
Low	-1.10	2.33	-1.72	0.36	-1.55	-0.35
	(-3.49, 1.29)	(-0.96, 4.75)	(-3.80, 0.36)	(-2.53, 3.25)	(-4.21, 1.11)	(-2.50, 1.81)
Medium	-0.85	1.77	1.52	1.04	-1.00	-1.12
	(-2.43, 0.74)	(-0.32, 3.87)	(-0.78, 3.84)	(-0.86, 3.00)	(-3.02, 1.02)	(-3.08, 0.62)
High	Ref	Ref	Ref	Ref	Ref	Ref
**Cigarettes/day**						
1–10	1.14	0.67	0.20	4.62	2.66	2.02
	(-4.03, 6.17)	(-1.09, 2.44)	(-2.88, 3.29)	(-1.08, 10.32)	(-3.44, 8.76)	(-1.96, 6.01)
11–20	-0.65	0.37	-0.09	3.32	1.93	2.68
	(-4.05, 6.34)	(-1.20, 1.94)	(-3.01, 2.84)	(-2.50, 9.14)	(-4.19, 8.05)	(-1.20, 6.56)
21–30	0.98	-0.73	-0.58	3.60	2.73	3.86*
	(-4.26, 6.24)	(-2.67, 1.20)	(-4.01, 2.84)	(-2.27, 9.48)	(-4.17, 9.65)	(0.22, 7.71)
>30	Ref	Ref	Ref	Ref	Ref	Ref

Missing cases were excluded from the analysis. Ref: indicates the reference group in the linear regression analyses.

* p<0.05

** p<0.01

*** p<0.001.

### Effects of warning labels

The proportion of smokers who reported noticing warning labels ‘often or very often’ varied across countries (Table 5). Specifically, rates were lowest in Greece and Hungary, and highest in Romania and Poland where more than half of respondents reported they noticed the warnings. Less than one in five smokers declared that they read the warning labels often or very often, except in Romania where one-third of the smokers declared this.

**Table 5:** Effects of warning labels and Label Impact Index (LII) scores overall and by country (n=6011)

*Variable*	*Overall*	*Germany*	*Greece*	*Spain*	*Romania*	*Hungary*	*Poland*
	*% (SE)*	*% (SE)*	*% (SE)*	*% (SE)*	*% (SE)*	*% (SE)*	*% (SE)*
Noticing	38.3 (1.1)	30.7 (2.7)	21.4 (2.4)	36.6 (3.0)	56.5 (2.8)	25.2 (2.6)	59.2 (2.9)
Reading	20.2 (0.9)	11.5 (1.5)	21.9 (2.5)	12.6 (1.2)	37.6 (2.9)	15.0 (2.3)	20.7 (2.4)
Thoughts	5.6 (0.4)	4.7 (1.2)	5.1 (1.0)	5.1 (1.0)	12.8 (1.3)	3.0 (0.8)	3.3 (0.9)
Quitting	4.0 (0.4)	3.2 (0.9)	2.5 (0.7)	2.4 (0.6)	10.6 (1.3)	1.4 (0.5)	3.9 (1.0)
Foregoing	15.4 (0.8)	10.3 (1.5)	13.5 (1.7)	14.2 (2.2)	14.5 (1.7)	18.3 (2.3)	21.6 (2.6)
Avoidance	13.3 (0.7)	9.9 (1.4)	14.2 (1.6)	19.9 (1.9)	16.5 (2.0)	9.2 (1.8)	10.4 (1.6)
**Saw quitting supports**							
Helpline number^b^	24.0 (1.0)	28.6 (2.3)	6.5 (1.3)	19.2 (2.4)	36.3 (2.6)	21.8 (3.0)	31.4 (2.4)
Quitting website^b^	15.2 (0.8)	20.9 (2.1)	5.4 (1.1)	15.7 (1.7)	17.9 (1.9)	14.8 (2.3)	16.4 (1.9)
**Health Information**							
Want more	14.4 (0.7)	8.4 (1.3)	23.5 (2.1)	14.9 (1.7)	25.1 (2.2)	3.0 (0.6)	11.9 (1.3)
Want the same	63.1 (1.1)	46.5 (3.0)	63.8 (2.7)	61.2 (2.3)	60.6 (2.7)	72.2 (2.7)	74.3 (2.1)
Want less	18.3 (0.9)	40.0 (3.0)	10.9 (1.7)	18.8 (2.0)	11.1 (1.3)	21.9 (2.5)	7.3 (1.4)
	**Mean Score (SE)**						
Label Impact Index (LII)	-0.071 (0.147)	-1.090 (0.327)	-0.335 (0.364)	-0.378 (0.318)	1.519 (0.343)	-0.650 (0.378)	0.507 (0.422)

Label Impact Index (LII) is a composite measure that weights and standardizes scores for four of the six label effects indicators based on non-dichotomized data (i.e. original four/five-point scales). LII = (NOTICING × 1) + (THOUGHTS × 2) + (QUITTING × 2) + (FORGOING × 3). Higher scores indicate greater impact.

The percentage of smokers who recalled seeing a helpline number on cigarette packs was around 30% in Germany, Romania and Poland, but was lower than 22% in Hungary and Spain, while in Greece it was around 7%. Information regarding quitting websites was noticed on cigarette packs by about 15% of smokers in all countries, ranging from the highest in Germany (20.9%) to the lowest in Greece (5.4%).

The percentage of smokers who declared that the warning labels made them think ‘a lot’ about health risks or made them more likely to quit was less than 6% for all countries, except in Romania, where more than 10% declared this. In Germany, Greece, Spain and Romania, 15% or less of smokers declared that in the last 30 days warning labels made them refrain from smoking at least once, while the percentage in Hungary and Poland was around 20%.

LII scores were lowest in Germany and Hungary, followed by Greece and Spain, and highest in Poland and Romania (Table 5). In all countries, except for Spain, the LII was higher among respondents who reported lower daily cigarette consumption (Table 6). In Poland, the LII was lower among people with lower educational level, while in Romania it was higher among those with low income. In Poland and Romania, it was lower among younger age groups, while in Greece a reverse situation was found. In Germany, urban residence was positively associated with a higher LII, while in Poland, the LII was lower among men.

**Table 6:** Predictors of the Label Impact Index (LII) (results of linear regression analysis)

*Variable*	*Germany*	*Greece*	*Spain*	*Romania*	*Hungary*	*Poland*
	*B (95% CI)*					
**Age group**						
18–24	1.11	-0.20	1.01	-2.27**	-1.08	0.07
	(-0.29, 2.52)	(-1.88,1.49)	(-1.44, 3.45)	(-3.69, -0.84)	(-3.45,1.28)	(-2.28, 2.42)
25–39	0.50	1.52*	-1.09	-0.82	1.14	-1.31*
	(-0.58, 1.58)	(0.27, 2.78)	(-2.47, 0.30)	(-2.25, 0.61)	(-0.30, 2.59)	(-2.34, -0.27)
40–54	0.40	1.30	-0.05	0.46	-0.26	-0.41
	(-0.44, 1.24)	(-0.09, 2.70)	(1.66, 1.56)	(-0.90, 1.82)	(-1.61, 1.09)	(-1.68, 0.85)
> 54	Ref	Ref	Ref	Ref	Ref	Ref
**Gender**						
Male	-0.57	-0.02	-0.91	-0.64	-0.61	-1.19**
	(-1.20, 0.06)	(-0.83, 0.80)	(-1.88, 0.06)	(-1.67, 0.40)	(-1.35, 0.14)	(-1.95, -0.43)
Female	Ref	Ref	Ref	Ref	Ref	Ref
**Urbanization degree**						
Urban	1.66*	-1.13	-0.51	-0.73	0.11	0.97
	(0.16, 3.17)	(-3.57, 1.32)	(-3.14, 2.13)	(-2.13, 0.67)	(-1.95, 2.17)	(-0.93, 2.88)
Semi-urban	0.23	-0.25	0.46	0.05	-0.310	0.34
	(-1.08-1.54)	(-2.06, 1.55)	(-2.11, 3.03)	(-1.71, 1.81)	(-2.26, 1.64)	(-1.68, 2.36)
Rural	Ref	Ref	Ref	Ref	Ref	Ref
**Education**						
Low	-1.17	-1.05	-0.38	-0.88	-1.45	-1.97*
	(-2.90, 0.57)	(-2.53, 0.42)	(2.46, 1.70)	(-2.63, 0.88)	(-4.27, 1.36)	(-3.91, -0.03)
Medium	-1.19	-0.30	0.38	-0.31	-1.36	0.71
	(-3.16, 0.79)	(-1.58, 0.98)	(-1.59, 2.34)	(-1.82, 1.20)	(-4.26, 1.52)	(-0.87, 2.29)
High	Ref	Ref	Ref	Ref	Ref	Ref
**Income**						
Low	-0.05	0.94	0.74	1.68*	0.87	-0.01
	(-1.28, 1.18)	(-0.50, 2.39)	(-1.04, 2.52)	(0.18, 3.18)	(-0.76, 2.50)	(-1.88, 1.87)
Medium	-0.05	1.29	1.41	0.86	1.22	-0.17
	(-0.94, 0.84)	(-0.07, 2.66)	(-0.28, 3.10)	(-0.27, 2.00)	(-0.21, 2.66)	(-1.50,1.17)
High	Ref	Ref	Ref	Ref	Ref	Ref
**Cigarettes/day**						
1–10	2.90**	1.83**	1.80	2.97*	4.27**	2.91**
	(1.14, 4.65)	(0.49, 3.18)	(-0.16, 3.75)	(0.03, 5.92)	(1.78, 6.75)	(1.19, 4.63)
11–20	1.46	0.93	0.95	1.01	2.28	2.76**
	(-0.05, 2.97)	(-0.18, 2.04)	(-0.88, 2.79)	(-1.67, 3.70)	(-0.27, 4.58)	(1.17, 4.35)
21–30	0.92	0.24	0.32	-0.39	1.26	1.49
	(-0.70, 2.55)	(-1.47, 1.94)	(-1.77, 2.40)	(-3.25, 2.49)	(-1.89, 4.43)	(-0.63, 3.60)
>30	Ref	Ref	Ref	Ref	Ref	Ref

Missing cases were excluded from the analysis. Ref: indicates the reference group in the linear regression analyses.

* p<0.05

** p<0.01

***p<0.001.

Around two-thirds of smokers reported they wanted the same amount of health information to appear on cigarette packages, with the exception of Germany where more than 40% of smokers reported wanting less information on warning labels.

### Relationship between the level of knowledge and labels impact index

Table 7 shows that in all countries, except for Poland, a higher LII score was positively associated with a higher KHR index score, even after controlling for sociodemographic variables and number of cigarettes smoked per day. Higher LII scores were also associated with greater knowledge of specific smoking-related diseases and conditions.

**Table 7:** Relationship between knowledge of health effects (KHR index as well as specific knowledge about several health risks) and label impact index (LII)

*Variable*	*Germany*	*Greece*	*Spain*	*Romania*	*Hungary*	*Poland*
	*B (95% CI)^a^*					
KHR Index	0.33***	0. 15**	0.29***	0.30***	0.30***	0.14
	(0.18, 0.49)	(0.04, 0.25)	(0.16, 0.42)	(0.20, 0.40)	(0.13, 0.46)	(-0.03, 0.30)
**Health Effect**	**Adjusted OR (95% CI)^b^**					
**Active smoking**						
Heart disease	1.10***	1.02	1.16***	1.16***	1.05*	0.99
	(1.04, 1.15)	(0.96, 1.09)	(1.07, 1.26)	(1.08, 1.23)	(1.01, 1.11)	(0.93, 1.07)
Impotence	1.08**	1.05*	1.08**	1.07***	1.06**	1.06**
	(1.03, 1.12)	(1.01, 1.09)	(1.02, 1.14)	(1.04, 1.10)	(1.02, 1.10)	(1.01, 1.11)
Lung cancer	1.10*	1.21*	1.07	1.08	1.00	1.00
	(1.01, 1.18)	(1.03, 1.41)	(0.97, 1.18)	(1.00, 1.17)	(0.92, 1.05)	(0.93, 1.05)
Blindness	1.09***	1.02	1.07***	1.07***	1.07**	1.08***
	(1.04, 1.14)	(0.99, 1.06)	(1.04, 1.12)	(1.04, 1.11)	(1.02, 1.11)	(1.05, 1.11)
Mouth cancer	1.08*	1.05	1.14**	1.10***	1.02	1.02
	(1.02, 1.14)	(1.00, 1.11)	(1.06, 1.23)	(1.05, 1.16)	(0.98, 1.07)	(0.97, 1.08)
Throat cancer	1.11**	1.05	1.08**	1.09**	1.03	0.99
	(1.05, 1.18)	(1.00, 1.13)	(1.02, 1.14)	(1.03, 1.16)	(0.98, 1.08)	(0.95, 1.05)
Stroke	1.06*	1.04	1.06**	1.11***	1.05*	1.02
	(1.01, 1.11)	(0.99, 1.09)	(1.01, 1.10)	(1.07, 1.15)	(1.01, 1.09)	(0.97, 1.06)
Emphysema	1.05	0.97	1.03	1.07***	1.05*	1.00
	(0.99, 1.09)	(0.91, 1.02)	(0.98, 1.09)	(1.04, 1.11)	(1.01, 1.09)	(0.95, 1.05)
Bronchitis	1.03	0.99	1.07	1.11***	1.04	1.05
	(0.97, 1.09)	(0.95, 1.05)	(0.98, 1.19)	(1.06, 1.16)	(0.99, 1.09)	(1.00, 1.10)
**Secondhand smoke**						
Lung cancer	1.06*	1.04	1.07**	1.05*	1.05*	1.04
	(1.02, 1.11)	(0.99 -1.09)	(1.01, 1.12)	(1.01, 1.09)	(1.01, 1.09)	(1.00, 1.09)
Heart attacks	1.07**	1.04	1.06*	1.07***	1.06**	1.06**
	(1.03, 1.12)	(0.99, 1.08)	(1.01,1.11)	(1.04,1.12)	(1.02, 1.10)	(1.01, 1.10)
Asthma in children	1.08**	1.05*	1.07*	1.10***	1.03	1.03
	(1.03, 1.13)	(1.10, 1.11)	(1.02, 1.13)	(1.06, 1.14)	(0.99, 1.07)	(0.99, 1.08)

Missing cases were excluded from the analysis.

a Linear regression analysis adjusted for age, gender, urbanization degree, income, education level, and cigarettes smoked/day.

b Multiple logistic regression analysis adjusted for age, gender, urbanization degree, income, education level, and cigarettes smoked/day.

* p<0.05

** p<0.01

*** p<0.001.

## DISCUSSION

### Principal findings

The findings of this study indicate that there are significant gaps in smokers’ understanding of the health risks of smoking across the six EU MS of the EUREST-PLUS ITC Survey. The vast majority of smokers were aware that smoking causes illnesses such as heart disease, lung and throat cancers. However, more than 20% of the smokers did not know that tobacco use causes mouth cancer, pulmonary diseases, and stroke. Smokers’ knowledge of the causal relationship between smoking and impotence and blindness was significantly lower. Importantly, with the exception of Romania, modest levels of knowledge of the health harms caused by exposure to secondhand smoke were observed across all six EU MS. These findings are similar to that of previous ITC reports for other countries^4,19-21^. All countries in the present study included health warning labels that address risk of heart attack, stroke, lung cancer, and impotence, and to a lesser extent messages regarding blindness, throat, or mouth cancers. Warning labels included non-specific or general messages in terms of lung disease but did not include specific messages related to emphysema or bronchitis. For the most part, warning labels did not include specific messages about health effects of secondhand smoke on heart disease and lung cancer or asthma in children. In general, levels of knowledge observed in countries for specific diseases and conditions corresponded to the specific warning labels’ messages in circulation.

There were several country-level differences documented, with Romania and Greece having a greater percentage of smokers who recognized the smoking health harms for most of the diseases/ conditions assessed and higher scores on the KHR index. The lowest KHR index scores were documented in Germany and Hungary. These trends did not necessarily correspond to strong health warning labels in these countries in 2016. Given that there are other sources of information on the health risks of smoking, including educational programs in schools, mass media, and medical advice, the higher rates of KHR scores documented in some countries are potentially the consequence of several activities, in addition to warning labels, however this requires further validation^22-24^. For instance, EUREST-PLUS Project findings show that there is an association between high exposure to mass media anti-smoking campaigns and increased knowledge of smoking health harms^25^. Moreover, in Hungary, despite the fact that pictorial warnings were used on tobacco packages, many smokers were using roll-your-own (RYO) tobacco, and packaging of RYO rolling papers and filters, which had no health warning information prior to August 2016^26^.

Several sociodemographic factors were associated with KHR index scores although trends varied between countries sampled. In three countries (Germany, Hungary, Poland) women had higher KHR scores, while in Romania and Greece, older age was associated with better KHR scores.

Our study found that only about one-third of smokers noticed warning labels often or very often, and only 20% read them often or very often. Approximately 5% of smokers declared that the warning labels made them think ‘a lot’ about health risks, or made them more likely to quit, and 15% reported warning labels made them refrain from smoking at least once. Seeing quitline and website information was reported at significantly lower rates in Greece, which is expected given this information did not appear on the warning labels in Greece in 2016 due to the non-existence of a National quitline.

Additionally, there was variability between countries with regard to health warnings’ impact on perceptions and actions of the smokers. For instance, Romania and Poland were the countries with the highest LII scores, while Germany and Hungary had the lowest scores. One potential explanation of the low LII score observed in Hungary could be that almost half of smokers in Hungary reported using roll-your-own (RYO) tobacco, while packaging of RYO rolling papers and filters had no health warning information prior to August 2016^26^. At the same time, the impact might vary between countries also as a consequence of type (only text or text and pictorials) and dimension of the health warning, and the type of messages^2-9^.

There was a correlation between higher daily cigarette consumption and lower LII scores. Other socioeconomic characteristics also influenced the LII at the country level. In Poland and Romania, the LII was higher among older age groups, while in Greece a reverse situation was observed. In Germany, urban residence was positively associated with a higher LII, while in Poland, the LII was higher among smokers with higher educational level. Other country-level factors, including the prevalence of tobacco use, characteristics of tobacco users and the extent to which comprehensive tobacco policies have been implemented are also expected to play a role in warning label effectiveness. As has been reported by others, it is possible that cultural factors, including receptivity to regulation, may play a role in explaining part of the differences observed between countries^27,28^.

In almost all countries, a significant positive relationship was documented between a KHR and LII scores. Romania and Germany being the countries where this relationship was evident for 10 or 11 of the 12 investigated health risks, while in the other countries the LII scores were associated with the level of knowledge for at least three health risks.

### Implications to practice

These data provide evidence to support the need for stronger education and policies that can enhance the effectiveness of health warnings among EU MS. Previous ITC studies showed that health warnings on cigarette packages are an important source of information regarding health effects of smoking^8,10^. A recent systematic review showed that stronger warnings increased attention to warnings, recall of warnings, and thinking about the health risks of smoking, as well as perceptions that warnings reduce smoking and motivate quitting^27^. There is substantial evidence to suggest that strengthening health warnings, such as those outlined in the EU TPD, will increase the effectiveness of warning labels and address some of the gaps in knowledge and salience, as well as promote quitting-related behaviors^2,3,5,27^. The documented gaps in knowledge regarding the health effects of secondhand smoke may also be an important target for public awareness in countries that are still struggling with implementation of comprehensive smoke-free public spaces, cars, and homes.

Importantly, this study serves as a baseline for examining the impact of the TPD, which began its implementation in most countries examined in the EUREST-PLUS ITC Wave 1 Survey in 2016. Future longitudinal studies should investigate changes in knowledge regarding health risks of smoking and the effectiveness of warning labels over time and as a consequence of implementing the TPD in the EU, which will be done within the context of this Horizon 2020 project.

### Limitations and strengths

While this study has many strengths with a large sample size of smokers across six nationally representative EU MS, findings should be interpreted in light of study limitations. First, the study used a cross-sectional design, which allows for the exploration of association rather than causality. The study included only current smokers and as such does not reflect individuals who recently quit smoking. The results are also based on participant self-report that may be subject to respondent and measurement bias. The present study examined variables prior to the implementation of the EU TPD and in some countries large pictorial warnings had been introduced just prior to our assessment and may not be sufficient to measure the effects of warning label changes in these countries. Likewise, KHR may have been influenced by other sources of education including mass media, which were not examined as part of this study. Given that the above warnings differed across countries including the size and health effects featured, some country level differences would be expected that were not controlled for in the analysis. In addition, there are other individual-level factors (e.g. nicotine addiction, presence of smoking-related illness or mental health illness) that might influence outcomes of interest that were not examined as part of this study.

Nevertheless, this study is the only one of its kind to provide a comprehensive baseline of the impact of health warning labels on smokers’ knowledge and smoking behaviors across multiple EU MS prior to the full implementation of the EU TPD, which will then be used to evaluate the impact of health warning provisions in a post-TPD assessment.

## CONCLUSIONS

This study has documented important gaps in EU smokers’ knowledge of the health effects of active and secondhand smoke and the impact of health warning labels prior to implementation of the EU TPD. These data provide evidence to support the need for stronger education and policies that can enhance the effectiveness of health warnings among EU MS, and will serve as a baseline for examining changes in these indicators following implementation of the TPD.

## CONFLICTS OF INTEREST

The authors declare that they have no competing interests, financial or otherwise, related to the current work. L. M. Lotrean reports grants from Aer Pur Romania, during the conduct of the study.

K. Przewoźniak reports grants and personal fees from the Polish League Against Cancer, outside the submitted work. C. I. Vardavas reports that he is the Strategic Development Editor of TID and that there are no conflicts of interest with this current work. The rest of the authors have also completed and submitted an ICMJE form for disclosure of potential conflicts of interest.
